# Role and Tasks of the Occupational Physician during the COVID-19 Pandemic

**DOI:** 10.3390/medicina57050479

**Published:** 2021-05-12

**Authors:** Lorenzo Spagnolo, Luigi Vimercati, Antonio Caputi, Marcello Benevento, Luigi De Maria, Davide Ferorelli, Biagio Solarino

**Affiliations:** 1 Section of Legal Medicine, Interdisciplinary Department of Medicine, University of Bari, Piazza Giulio Cesare 11, 70124 Bari, Italy; lorenzospagnolo90@gmail.com (L.S.); marcello.benevento@uniba.it (M.B.); davide.ferorelli@uniba.it (D.F.); biagio.solarino@uniba.it (B.S.); 2 Section of Occupational Medicine, Interdisciplinary Department of Medicine, University of Bari, Piazza Giulio Cesare 11, 70124 Bari, Italy; antonio.caputi@uniba.it (A.C.); luigi.demaria@uniba.it (L.D.M.)

**Keywords:** occupational medicine, COVID-19, SARS-CoV-2, risk assessment, risk management

## Abstract

*Background and Objectives:* The first clusters of SARS-CoV-2 infection were identified in an occupational setting, and to date, a significant portion of the cases may result from occupational exposure; thus, COVID-19 should also be considered a new occupational risk that both directly and indirectly impacts the health of workers. Given the significance of occupational-exposure-related infections and deaths, this study aims to assess the roles and tasks of occupational physicians (OPs) in countering the spread of the infection. Indeed, despite the OP’s centrality in risk management in the workplace, its activity in the current epidemic context has rarely been mentioned. *Materials and Methods:* Three different databases (PubMed, Google Scholar, and Embase) were questioned using the main keywords “COVID-19” and “SARS-CoV-2” that were crossed, according to different needs, with the terms “occupational medicine”, “occupational physician”, “workplace”, and “risk assessment” using, when possible, the MeSH database research. Additionally, a systematic research of the regulatory changes of workplaces health surveillance was performed on reference sites of international, European, and Italian authorities. *Results:* Fundamental tasks and duties of OPs in the current COVID-19 outbreak are highlighted by examining their clinical activity and technical action. A risk assessment and management workflow is proposed, and medico-legal implications in case of infection at work are also discussed in the light of recent regulatory changes that clearly attribute to OPs an important role in safeguarding public health. *Conclusion:* The proposed approach can provide new instruments to contrast the spread of the infection as part of a comprehensive system response to the current pandemic, for which OPs are called to assume full responsibility.

## 1. Introduction

On 31 December 2019, the World Health Organization (WHO) was notified of the detection of cases of unusual pneumonia of unknown origin in Wuhan City, the capital of Hubei Province in China. The first clusters of viral infection were identified in an occupational setting and specifically among workers of the Wuhan seafood market who accounted for more than half of the 47 cases detected until 1 January 2020 when the market was finally closed [1]. The virus was identified as a coronavirus and named SARS-CoV-2, responsible for COVID-19 disease. Given the quite high value of the basic reproductive number (R0) of 2.2, the virus rapidly spread all over the world to the point that on 30 January 2020, the WHO declared COVID-19 to be a public health emergency of international concern, and on 11 March 2020, it was declared a pandemic [2,3,4].

In the U.S., according to Baker et al., approximately 10% of workers are employed in occupations where exposure to disease or infection by biological agents occurs at least once per week. The majority of these are health care workers (HCWs), but high percentages of exposed subjects are also present in other occupational settings [5]. According to a recent technical report by the European Centre for Disease Prevention and Control (ECDC), a total of 1377 clusters of SARS-CoV-2 infection were reported in various occupational settings, including 18,198 COVID-19 cases. Among these, approximately 50% occurred in health and social care facilities, 21% occurred in the food packaging and processing industry, and 29% occurred in other settings [6].

Centers for Disease Control and Prevention (CDC) states that SARS-CoV-2 infection involves HCWs for at least 11% [7]. In Spain, a hospital reported similar percentages, as 11.1% of its 1911 employees tested positive for the virus [8]. In the UK, a comprehensive HCW screening program performed in a large Cambridge hospital pointed out a positivity rate for SARS-CoV-2 among HCWs of 3% in the asymptomatic screening arm and that of 15.4% in the symptomatic screening arm [9]. A study in Sweden assessed the seroprevalence of IgG antibodies against SARS-CoV-2 that was 19.1% among the 2149 HCWs working in a large acute care hospital [10].

In the University Hospital of Bari (Apulia Region, Southern Italy), the enhancement of preventive measures, the design of a reporting system, and the implementation of measures of preventive isolation (COVID-19 Hotel) reduced the prevalence of infection among HCWs [11,12,13,14].

Currently, in the world, the virus has affected approximately 152 million people, with approximately 2800 deaths of HCWs among a total of approximately 300,000 reported infections, according to a recent survey [15]. To date, Italy has had more than 100,000 deaths due to COVID-19, with a significant portion of them attributable to occupational exposure, especially referring to HCWs.

Thus, COVID-19 should also be considered a new occupational risk that impacts workers’ health both directly and indirectly and further fuels the spread of the pandemic in the community.

Given the significance of occupational-exposure-related infections and deaths, not only from a numerical, but also from a strategic point of view, this study aims to assess the role and tasks of occupational physicians (OPs) in countering the spread of the infection. Indeed, despite the OP’s centrality in risk management in the workplace, its activity in the current epidemic context has rarely been mentioned. 

The main research questions of the study are:

1. What are the fundamental tasks and duties of OPs in the current COVID-19 outbreak considering the recent regulatory changes?
2. How the OP’s clinical activity and technical action should be carried out?
3. What are the main medico-legal implications in case of infection at work?

## 2. Materials and Methods

### 2.1. Literature Search

For the identification process of relevant sources, we conducted a two-step literature search. As a first step, two investigators (L.S. and M.B.) independently questioned three different databases (PubMed, Google Scholar, and Embase) using the main keywords “COVID-19” and “SARS-CoV-2” that were crossed, according to different needs, with the terms “occupational medicine”, “occupational physician”, “workplace”, and “risk assessment” using, when possible, the MeSH database research. The following strings were used:

- (“COVID-19” OR “SARS-CoV-2”) AND (“occupational medicine” OR “occupational physician”);
- (“COVID-19” OR “SARS-CoV-2”) AND “workplace”;
- (“COVID-19” OR “SARS-CoV-2”) AND “risk assessment”.

Additionally, a systematic research of the regulatory changes of workplaces health surveillance was performed on reference sites of international, European, and Italian health and government authorities. 

**Figure 1:**
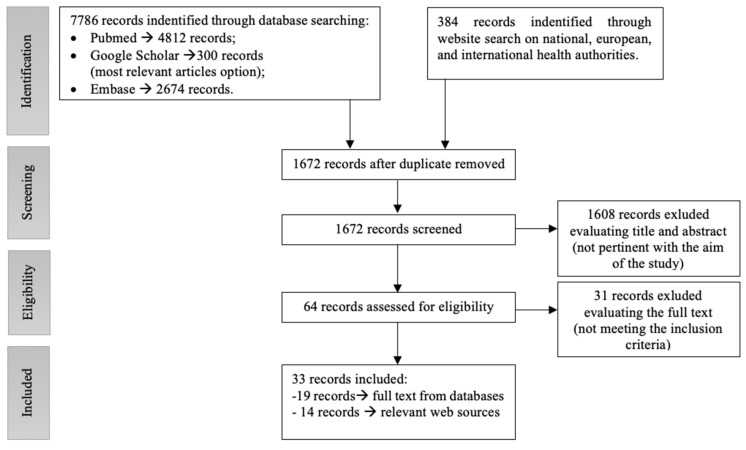
Review flow-chart and result summary.

Papers and web sources identified were screened for duplicate removal, and remaining records were examined by the evaluation of both the title and the abstract and excluded if judged not pertinent with the aim of the study. The subsequent step involved the implementation of an additional search based on the reference list of the retrieved paper by another independent reviewer (A.C.). Relevant articles and web sources were then evaluated for eligibility by two different investigators (D.F. and LD.) based on a full-text reading (Figure 1). 

### 2.2. Inclusion and Exclusion Criteria

Papers and web sources were included if dealing with the role and tasks of OPs and with their activity in risk assessment and management during the COVID-19 epidemic. We excluded papers written in languages other than English and Italian, and those papers of which the full texts were unavailable even after contacting the corresponding author.

## 3. Results

We obtained a total of 7786 records from the referral scientific databases (Pubmed, Google Scholar, and Embase) and further 384 records on web platforms. After duplicates, removal and screening of 1608 nonrelevant articles records were excluded, while the remaining 64 were assessed for eligibility. Thirty-three articles/web sources were included in the study, which met the inclusion criteria. The process of identifying eligible sources is outlined in Figure 1.

## 4. Discussion

### 4.1. Roles and Tasks of OPs during the COVID-19 Crisis

The COVID-19 crisis triggered extraordinary measures that played an important role in the development of labor laws in many countries. The major objectives of those choices are to reduce the risk of infection in the workplace, to alleviate the financial consequences for businesses and workers and to ensure the performance of essential work [16,17]. The risks of infection in the workplace especially gave rise to numerous discussions on the preventive measures to be taken according to different settings and jobs, and the respective roles of company managers, employees, or the labor inspectorate are widely debated. The role of OPs, although fundamental in this pandemic context, is rarely mentioned [18].

Nevertheless, OPs are essential professional figures among multidisciplinary occupational health teams. The training and fundamental competencies of OPs have evolved over time to adequately react to incessant changes in working conditions and to meet the needs of society as a whole. The WHO European Centre for Environment and Health (ECEH) defined OPs as medical specialists “dealing with the assessment of workers’ health, linking working conditions and processes to workers’ health, assisting in managing the health, skills and working capacity of the entire working population and managing individual cases in the context of working ability and production”; therefore, coping “with primary, secondary and tertiary prevention of ill health in the workforce, with a potential influence on the health of the population as a whole” [19]. Moreover, the ECEH assessed the fundamental clinical knowledge and skills of OPs, among which worth mentioning in light of the recent epidemic are the following:

- The identification, management, and prevention of infectious diseases (including zoonosis);
- The diagnostics, management, and referral (where necessary) for specialist assessment and treatment in case of respiratory disease.

However, despite this general attribution of tasks and competencies, the roles and responsibilities of OPs vary from country to country in relation to national laws; thus, occupational health and medical services are not evenly structured around the world, reflecting important differences in health, social security and insurance delivery systems.

In Italy, indeed, the duties and responsibilities of OPs are defined by Legislative Decree n.81 of 9 April 2008 [20]. Among law-defined obligations incumbent on the occupational physician, a fundamental obligation is planning and performing health surveillance through health protocols defined according to specific working risks and considering the most advanced scientific guidelines. The law, therefore, affirms the obligation for OPs to take an active role in planning, as well as carrying out, the required health surveillance through health protocols calibrated on specific risks in relation to the particular job performed by the worker, thus avoiding all those broad-spectrum assessments not aimed at their prevention.

The current epidemic has, therefore, posed a problem regarding the responsibility of OPs, in light of their strictly prophylactic role, in the prevention of new epidemic outbreaks in the workplace. Indeed, at the peak of its diffusivity, COVID-19 was and still is considered a generic risk equally incumbent in the entire population, except for HCWs who are directly exposed to infected patients. This issue was first addressed in the context of a protocol shared between social partners on 14 March 2020 for the regulation, countering and containment of the spread of COVID-19 in the workplace [21]. This document, once again, highlights the strategic importance of health surveillance, which unlike other elective health activities should not be interrupted and should take care of reintegration into the workplace of subjects who have overcome the infection. The role of identifying so-called “fragile subjects” is also attributed to OPs, who should identify those who, due to age or individual pathological conditions, may be more prone to contract the severe form of COVID-19.

The same principles were then reaffirmed in the subsequent paper, signed on the following 24 April (attached to Presidential Decree of 11 June 2020) which, taking a further step forward, assigns to OPs, in consideration of their preventive role in health surveillance, the task of suggesting the adoption of diagnostic tools (e.g., swabs and serological tests) if judged useful for containing the spread of the virus and for safeguarding the health of workers [21,22]. The resulting ministerial circular (29 April) also follows the same line, recalling the role of the OP in contact tracing through the early identification of close contacts in the workplace and their isolation with the collaboration of general practitioners and prevention departments [23]. 

### 4.2. COVID-19 Risk Assessment in an Occupational Setting

The mentioned Legislative Decree n.81 outlines, under Title X, the risk from exposure to biological agents. These are defined as “any microorganism, even if genetically modified, cell culture and human endoparasite that could cause infections, allergies or poisoning” and are divided into four groups as indicated by Directive 2000/54/EC [24]. Similarly, but with some relevant differences, four qualitative risk groups for biological agents were also established by the National Institutes of Health (NIH) and embraced by the American Industrial Hygiene Association (AIHA) in 2006 (Table 1) [25].

Before the recent epidemic, coronaviruses were located in group 2. However, in light of the clinical and epidemiological data of SARS-CoV-2, the European Commission decided to reallocate them to group 3 [26].

SARS-CoV-2 clearly requires a risk assessment in the occupational setting. The occupational risk assessment for biological hazards in the workplace, unlike other hazards (e.g., chemical hazards), is particularly challenging because of the high level of variability in exposures, sampling methods, differences in worker vulnerability, and a lack of epidemiological data to support reliable exposure limits [27].

Available industrial hygiene and environmental health decision-making frameworks designed to control nonbiological hazards in the workplace can be tailored to infectious diseases using the so-called Anticipate, Recognize, Evaluate, Control, and Confirm (ARECC) conceptual scheme [28].

This paradigm includes hazard assessments (identifying and reviewing hazard data; selecting hazard criteria) and exposure assessments (collecting exposure data via measurement and/or modelling), of which the combination leads to risk characterization and to the development of a risk management programme (developing programmes, hierarchy of controls and control banding; confirming effective implementation) [29]. However, in relation to the peculiar characteristics of SARS-CoV-2, this risk characterization cannot be exclusively based on the features of the working context, but also on the specific biological characteristics of the subject whose risk profile may be significantly different.

Therefore, to provide a global vision, a three-step approach to risk assessment has been proposed, consisting of [30]:

- Workplace risk (WR) assessment;
- Clinical vulnerability risk (CVR) assessment;
- Overall occupational health risk (OHR) assessment.

The WR assessment should be performed in advance of CVR assessment. Moreover, it is believed that in the OHR evaluation, conditions for reaching the workplace (RW) (e.g., use of public transport) and territorial and local characteristics of viral spread (LVS) cannot be neglected. For the latter, the estimation of the local Rt index (Real Time R0) should be considered.

**Figure 2:**
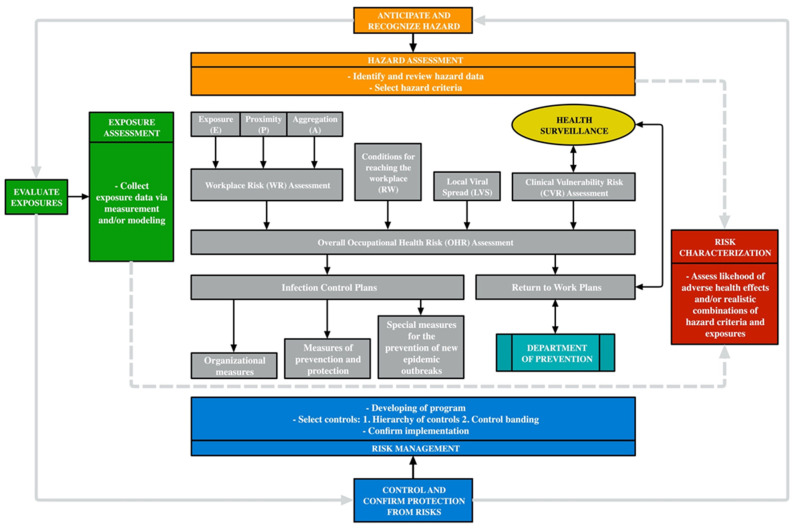
Summary of the proposed risk assessment and management workflow.

The interpolation of these risk assessments considered as a whole makes it possible to attribute to the individual worker a correct estimation of OHR and consequently allows the employer, in collaboration with OPs, to implement the risk management program and to perform all the necessary measures to minimize the risk for the health of workers (Figure 2).

#### 4.2.1. Workplace Risk Assessment

According to the U.S. Department of Labor Occupational Safety and Health Administration (OSHA), worker risk of occupational exposure to SARS-CoV-2 may range from very high to high, medium, or lower (caution) risk in relation to a pyramidal vision of the risk classes, which are independent of the assessment of individual clinical susceptibility [31].

Similarly, in light of recent regulatory changes, the Italian National Institute for Insurance Against Accidents at Work (INAIL) developed a technical document for WR assessment according to different working sectors and contexts. Operative indications for the remodeling of health surveillance have also been proposed [32]. The methodology used for risk assessment according to different working contexts is based on a model derived from the data of the O*NET 24.2 database adapted to the Italian working context as described by the INSuLa 2 survey and by the data of employed persons in 2019 provided by the National Institute of Statistics (ISTAT) [33,34]. 

Three variables are taken into account to classify the risk of infection in the workplace:

1. Exposure (E): the likelihood of coming into contact with sources of contagion in carrying out specific work activities (e.g., health sector, management of special waste, and research laboratories);
2. Proximity (P): the intrinsic characteristics of the job that do not allow sufficient social distancing (e.g., specific tasks in assembly lines) for part of working time or almost all;
3. Aggregation (A): the type of job that involves contact with other subjects other than the company’s workers (e.g., catering, retail trade, entertainment, hospitality, and education).

The first two variables (E and P) correspond to a different scale of values which are combined according to the production sector under consideration. The resulting score is then corrected with the scale corresponding to the last variable (A). The final result determines the attribution of the risk level with its color code for each production sector within a matrix. 

There is no doubt that the most difficult variable to characterize is exposure which is strictly dependent on the mode of transmission of the virus. The evidence currently available identifies the main transmission mechanism through large respiratory droplets (diameter: >5 μm) from direct contact with an infected subject. However, under well-defined conditions, airborne transmission through aerosols is also possible via droplets with diameters of <5 μm or “microdroplets” which can remain suspended in the air of environments for a long time. Transmission also appears possible through indirect contact with contaminated surfaces followed by touching the eyes, nose, or mouth (fomite transmission) [35].

A quantitative exposure evaluation method for direct-contact/droplet-transmission and airborne-transmission pathogens was proposed by Sietsema ed al., who based exposure (*E*) assessment on the likelihood (*L*) and duration (*D*) of contact with potentially infectious subjects according to the following equation: E=L×D [36]. This equation, however, does not apply to indirect surface contact transmission. 

#### 4.2.2. Clinical Vulnerability Risk Assessment

According to the norm, the concept of “fragility” concerns the health conditions of the worker with respect to pre-existing pathologies (two or more), which could determine, in case of infection, a more serious outcome or death, also regarding the risk of exposure to contagion [22]. Moreover, according to the CDC, workers at increased risk for severe illness are essentially identifiable within two categories: older adults and people with medical conditions [37].

Increased age represents a strong risk factor for severe COVID-19 outcomes, as epidemiological data clearly report a steady increase from 50 to 60 years of age. However, recent studies have shown that chronic age-dependent diseases could actually strongly influence this assumption. Indeed, the crude effect of age results substantially decreased when adjusting for important age-dependent pathologies (e.g., diabetes, hypertension, cardiovascular and cerebrovascular diseases, immunodeficiency, COPD (Chronic Obstructive Pulmonary Disease), and renal disease) [38].

Therefore, CVR concerning the “biological” individual characteristics of the person should, above all, take into account the presence of pathological conditions that are proven to be positive predictors of the onset of a more severe clinical course.

However, the intrinsic variability of these diseases and their possible coexistence in a single individual do not allow a quick pursuit of a standardized score that, even if identified, would risk oversimplifying the risk profile assessment, not taking into account the real person. Therefore, a task of the physician should be considered that assesses CVR through health surveillance and OP clinical activity, which, taking into account all the factors mentioned above, attributes a high, medium, or low value to the risk.

#### 4.2.3. Overall Occupational Health Risk Assessment

**Figure 3:**
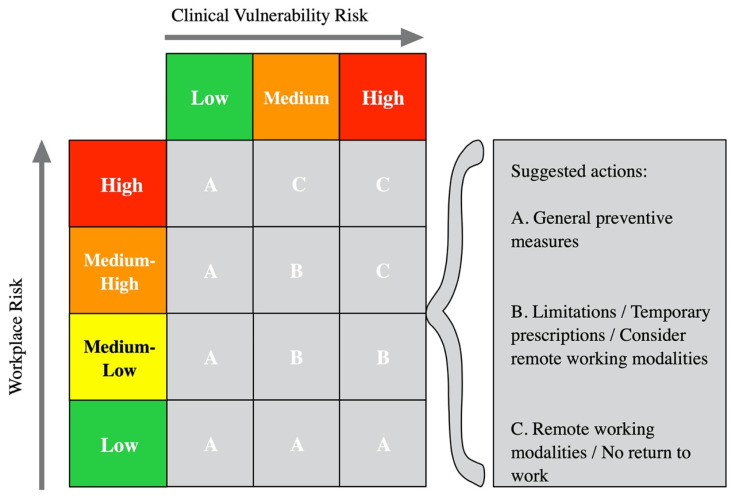
Interpretation of overall health risk (OHR) and suggested actions.

Thus, the overall interpretation of the data derived from WR and CVR allows reaching OHR by stratifying the risk classes into A (low risk), B (medium risk), and C (high risk) by using a risk matrix approach (Figure 3).

### 4.3. Risk Management in the Occupational Setting

After OHR is properly evaluated, a risk management strategy has to be implemented in order to mitigate the risk of transmission in the workplace. This includes infection control plans and return to work plans.

Referring to infection control plans, the National Institute for Occupational Safety and Health (NIOSH) hierarchy of controls (HCs) is also applied to NIOSH total worker health, and control banding is a concept and a tool that could be successfully tailored to the current pandemic [28,39,40].

HC refers to a mean of determining how to implement effective control solutions. The idea behind is that control methods are not equally effective so that those at the top of the hierarchy should be implemented first in order to pursue an inherently safer system, where the risk has been substantially reduced. Elimination and substitution methods are the most effective at reducing hazards but are also difficult to apply, especially referring to infectious diseases. Engineering controls are preferable over administrative and personal protective equipment (PPE) for controlling existing risks in the workplace, because they are designed to remove the hazard before it even comes in contact with the worker and independently of worker interactions. Instead, administrative controls and PPE are frequently used, when hazards are not well controlled. These methods are less effective, require significant effort by workers and, over the long term, can be very costly to sustain. 

Control banding is based on the hierarchy of controls but requires the prior assignment of the job to a specific risk category to which controls will be subsequently applied. Control measures will be thus modulated on the basis of the specific risk class and of the actual logistical possibilities of the company and the job performed that has to be considered in its specificity.

According to the INAIL technical document, a series of actions are suggested to be adopted to prevent/mitigate the risk of contagion for workers [32]. These measures should be included as a supplement to DVR (Document for Risk Evaluation) and can be classified as:

- Organizational measures;
- Measures of prevention and protection;
- Specific measures for the prevention of new epidemic outbreaks.

The organizational measures concern the following: (1) the remodeling of spaces and workstations to favor social distancing (compatibly with the nature of production processes); (2) the redefinition of working time which, if possible, should limit the simultaneous presence in the workplace through flexibility, the re-articulation of shifts, and the adoption of forms of remote work.

The measures of prevention and protection concern the following: (1) adequate and contextualized information and training-in times of “infodemic” sources for information must be the institutional ones, thus avoiding the multiplication of fake news; (2) hygiene and sanitation measures of the environments; (3) use of masks and PPE; (4) health surveillance and protection of “fragile” workers.

The specific measures for the prevention of new epidemic outbreaks that should be performed during the transition phase regard contact tracing operations also through the execution of nasal swabs and validated serological tests.

Return to work plans, instead, usually are made of decision frameworks which include a multiphase algorithm. The decision-making steps are obviously affected by national laws and protocols regarding both the time of isolation/quarantine and healing criteria (based on diagnostic or follow-up tests). In this scenario, the OP must necessarily interface with prevention departments. It is, therefore, desirable to create shared paths allowing establishing different skills and roles, so that both the speed of reintegration into work and a more effective contact tracing are granted (Figure 2).

### 4.4. Health Surveillance

Notwithstanding the new role assumed by OPs in contact tracing, there are two main changes in the field of health surveillance that concern the following: (1) the protection of so-called “fragile subjects” and (2) reintegration into the workplace of those who have recovered from the infection [22].

- For the protection of “fragile subjects”, the concept of “exceptional health surveillance” is introduced, which evaluates through a medical examination performed by the OP, also appointed for this very purpose, and can also be directly requested by the possibility of the worker expressing a judgement of temporary unfitness or limitations of fitness for an adequate period, with careful reassessment at its end;
- for the gradual reintegration of healed workers, the medical examination prior to resuming work to assess specific risk profiles has to be performed following the absence from the workplace in any case, regardless of the length of absence due to illness.

In light of the increased risk of contagion and of the strategic importance obviously covered, health surveillance in health and hospital facilities must guarantee tight control and close monitoring of contacts. The aim is to limit the spread of the infection among HCWs and to guarantee the safety of patients and of health care as a whole.

### 4.5. Liability and Medico-Legal Issues

Although still in an emergency condition, this new and central role assumed by the OP in the context of this “system” response is not free from control by the judicial authority. In fact, this assumption of responsibility could lead to the formation of profiles of penal liability, although in a purely theoretical line. These regard the following:

- the missed recognition of workers with biological risk profiles that configure a condition of fragility: in the occurrence of infection in the workplace that results in death or personal injury, the OP could be prosecuted for the related offenses (art. 589–590 of the Italian penal code);
- in case of failure to adopt the appropriate measures for contact tracing: in the occurrence of the explosion of epidemic outbreaks in the workplace, the OP could be prosecuted for the crime of culpable epidemic (art. 438–452 of the Italian penal code).

Moreover, another central medico-legal issue is the issue relating to compensation in the occurrence of contagion in the workplace. Indeed, in Italy, as of 15 May 2020, compensation claims for supposed workplace-acquired SARS-CoV-2 infection registered by INAIL were 43,399, equal to 19.4% of the total cases and approximately 30% of people of working age [41].

The doctrine in this case is divided between those who argue that, due to its particular characteristics (fortuitous, violent, and external cause), SARS-CoV-2 infection should be considered an accident at work, and those who argue that it should be compensated in the same way as an occupational disease [42,43]. 

The former interpretation is prevalent in Italy as stated also by the INAIL, which specifies in Circular no. 22 of 20 May 2020 that infectious diseases resulting from an occupational exposure (not only COVID-19, but also hepatitis, brucellosis, AIDS, tetanus, etc.) have always been classified and treated as an accident at work since the virulent cause is equivalent to the violent cause, even when its effects become evident after a certain time [44].

The latter is, for instance, the one accepted in France, where COVID-19 has already been classified as an occupational disease and tabulated as such [16]. 

If this theme raises relevant problems that already now but even more in the future will generate high volumes in terms of litigation, the evaluation of the causal link appears even more problematic. Indeed, given the current diffusion of the virus and its high contagiousness, it is undoubtedly difficult to establish a causal link with work rather than with other nonwork “contagion opportunities”.

The debate is very hot on these topics, promising significant novelties in the short term.

## 5. Conclusions

At the time of the COVID-19 pandemic, OPs seem to have gained a key role in the context of a comprehensive “system” response in countering the spread of the infection. This legitimation in terms of role and tasks comes from both government and health authorities, which clearly attributes to OPs a key role in safeguarding public health through his technical action and his clinical activity. The first should be carried out through the estimation of the risk of contagion in the workplace, risk management, and implementation of protective measures, and the latter should be conducted through the protection of “fragile subjects” and reintegration into working activities after the infection. This effort to reorganize working activities ensuring workers’ safety and health is required to avoid the onset of new epidemic outbreaks and to provide a new preventative approach for which OPs are called to assume full responsibility. Indeed, this new central role implies relevant responsibilities from the penal and civil point of view, so that a correct management of the worker can contribute to an overall reduction of litigation and reimbursements for infections contracted in the workplace. 

## Figures and Tables

**Table 1:** Biological agent classification according to the American Industrial Hygiene Association (AIHA) and the National Institutes of Health (NIH) and to L.D. n.81-2000/54/EC.

Biological Agent Group	AIHA/NIH			L.D. n.81 and 2000/54/EC
	Individual Risk	Community Risk	Definition	Definition
**1**	Low	Low	Agents that are not associated with disease in healthy adult humans	Agent that is unlikely to cause human disease
**2**	Moderate	Low	Agents that are associated with human disease that is rarely serious; preventive or therapeutic interventions are often available	Agent that can cause disease and pose a risk to workers; unlikely to spread to the community; effective prophylactic or therapeutic measures are usually available
**3**	High	Low	Agents that are associated with serious or lethal human disease for which preventive or therapeutic interventions may be available (high individual risk but low community risk)	Agent that can cause serious illness and pose a serious risk to workers and can spread to the community; effective prophylactic or therapeutic measures are usually available
**4**	High	High	Agents that are likely to cause serious or lethal human disease for which preventive or therapeutic interventions are not usually available (high individual risk and high community risk)	Agent that can cause serious illness and pose a serious risk to workers and can present a high risk of spreading in the community; there are usually no effective prophylactic or therapeutic measures
